# 

**Published:** 1889-06-08

**Authors:** 


					150 THE HOSPITAL. June 8, 1889.
SPECIAL HOSPITAL SUNDAY NUMBER.
This Special Number will be issued on SATURDAY, JUNE 15th next,
and will consist of an issue of TWENTY THOUSAND Copies. It will
be sent as usual to every Clergyman and Minister of Religion who has a
Collection in aid of the Metropolitan Hospital Sunday Fund, and will be
distributed to the Congregations of many of the principal Church es and
Chapels. Applications for Free Copies for distribution should be made
at once by Clergymen and Ministers of Religion to the Publisher of The
Hospital, 139, Salisbury-court, E.C., the number of copies desired being
stated in the letter of application.
NOTICE TO CORRESPONDENTS.
All MS., Letters, Books for Review, and other matters intended for the
Editor, should be addressed The Editor, The Lodge, Porciiester
Square, London, W.
Notice to Advertisers and Subscribers.
All Advertisements, Orders for Copies of THE HOSPITAL, and Business
Communications should be addressed to The Publisher, not to the Editor
The Hospital, 139-140, Salisbury-court, Fleet-street, E.C.
Vol. V., now ready, 4s., post free. Cases for binding, may be had of
the Publisher, at Is. Cd. each.
To Residents, Secretaries, and Matrons.
The Editor will be glad to have the Regulations and each Report as
issued by Asylums, Hospitals, Convalescent and Nursing Institutions, as
well as those of other Charities, and an early copy in duplicate of all
Appeals and Festival Notices, etc., addressed to The Editor, The Lodge,
Porchestersquare, London, W. Any superintendent, secretary, matron,
or other chief official who regularly sends such information may be
registered, on application to the Publisher, to receive free of cost a cover
for binding The Hospital in half-yearly volumes.
Amusements and Relaxation.
Word Competition.
Commenced April 6th, Ends June 29th.
Ihree Prizes of 15s., 10s., 5s., will be given for the largest number of
-?words derived from the words set for dissection.
Proper names, abbreviations, foreign words, words of less than four
.letters, and repetitions are barred ; plurals, and past and present par-
ticiples of verbs, are allowed. Nuttail's Standard dictionary only to be
used.
N.B.?Word dissections must be sent in WEEKLY, arranged alpha-
betically, with correct total affixed.
The word for dissection for this, the Tenth week of the quarter, being
"CHURCHILL."
Results of Eighth Week.
Names. May 30th.
Patience  44
Nurse Duty   46
- Lightowlers   46
Scrooge   ?
Reldas  ?
linie   41
,K.G  38
Broxbourne   46
Lauriston   46
Spes  43
? Gretta  ?
Jb'rancesca   ?
Heatherbell .... ?
Sycamore   ?
Arctus   30
Amicus   45
. Scratcher   ?
Jemima   ?
See-Saw   46
Rosemont
-JennyWren ...! 39
Eric  23
Tiny  39
N'importe Qui .. 40
Names. May 30th
Pollie   28
W. G. R  26
Robe   ?
Beryl   29
Nesta   ?
Daffodil   ?
A Schoolgirl  ?
Montague   ?
Sheeny
Alice R
Belfast
Gentian   _
Hope
Louie
Hamilton
E. M  _
V. J. D  _
Ladyr
Surgical Nurse.. ?
L. Pearce   ?
Contentio  ?
M. W  46
Esperance  47
Notice to all Correspondents.
N.B.?All letters referring to this page which do not arrive at 139 and
140, Salisbury-court, London, E.C., by the first pest on Thursdays, and
?, are not addressed PRIZE EDITOR, will in future be disqualified and
v disregarded.
Scraps and Gleanings.
Diphtheria has again broken out at Sowerby Bridge.
Me. Clifford delivered his lecture on "Father Damien," at Bath
last week.
The Charity Organisation Society appeals for funds for its Con-
valescent Committee.
Dr. Arnold Lyndon lias been appointed house surgeon to the Royal
Orthopcedic Hospital.
The annual increase of the Esquimaux population is estimated to be
not more than 60 or 80.
Princess christian spoke in German when opening the Home for
German Seamen last week.
Some inhuman thieves lately stole the money in the collecting-box in
a vestibule at St. Thomas's.
Paddington Grebn Children's Hospital treated 423 in-patients
and 27,300 out-patients last year.
The cost of maintenance per head at Cork Hospital for Women and
Children last year was ?27. 5s. 5d,
An examination for 12 appointments as surgeon in the Indian Medical
Service, will be held on August 12th.
Milton, F.R.S., L.R.C.P., M.R.C.S., has been appointed assistant
house surgeon to St. Thomas's Hospital.
Dr. Beattie Crozier, author of " Civilisation and Progress," is a
medical practitioner in a London suburb.
Dr. Caffyn has written to the Daily Telegraph denouncing the
present out-patient system of our hospitals.
The Sanitary Committee of Norwich recommend the Town Council to
erect an isolation hospital at a cost of ?4,500.
Dr. Peyraud, of Vichy, claims to be able to cure hydrophobia by in-
jecting a virus derived from a herb called tansy.
The Egyptian Hospital has been closed for want of funds, and because
Port Said and Suez are considered sufficiently near.
Smallpox is rife amongst Captain Wissmann's troops at Zanzibar,
and, unluckily, the supply of vaccine lymph is very small.
The Drapers' Company have given ?25,000 to the People's Palace in
addition to their previous donations amounting to ?60,000.
The jubilee of the Royal Berks Hospital was celebrated by some
admirable theatricals given under distinguished patronage.
The Turner Medical Scholarship; has been awarded to Mr. T. W.
Kelynack, of Owens College and Manchester Royal Infirmary.
The next examination for the certificate in Psychological Medicine is
announced to take place in Bethlem Hospital on July 18th and 19th
next.
The arrangements for the annual street collection on Hospital
Saturday are now in hand, and many ladies have already promised
their aid.
Princess Christian will open the new buildings of the Cripples
Home and Industrial School for Girls, 17a, Marylebone-road, on Mon-
day, July 1st.
Presents of money are still being received for Murphy, the Dublin
leper, who has been removed to an isolated ward in the hospital and
made more comfortable.
Ms. George Archer, L.S.A., late medical officer of the Feltwell
District of the Thetford Union, has been granted a superannuation
allowance of ?50 per annum.
ABOUT 400 Red Cross men performed a sham rescue lately on suitable
ground near Berlin, saving the imaginary wounded from a battle-field
during and then after the battle.
Mr. Caleb Wright, M.P. for the Leigh division of Lancashire, has
signified his intention of contributing ?1,000 towards providing
recreation grounds for Tyldesley.
One of the staff of Bath Hospital most inhumanly refused to go and
see a man who was in a fit, because it was without his district. The
man died before another doctor could be found.
The late Mr. Robert Brewin has bequeathed ?2,500 for investment in
Government securities as the nucleus of funds to carry on his life-long
work for the prevention of cruelty to animals at Cirencester.
The Bishop of London, Mrs. Temple, and several well-known leaders
in temperance work, last evening attended the last public meeting in
connection with the annual fortnight's session of the Society of Friends.
The Court of Appeal has reserved judgment on the appeal of Dr.
Allbut, of Leeds, from a judgment against him of Baron Pollock in an
action brought by him against the General Council of Medical
Education.
A BOARD of officers who have been sitting at Portsmouth for a fort-
night, have reported that Clarence Barracks and Gunwharf Barracks
are in such an insanitary condition as to be dangerous to the health of
the troops.
In Manchester last week the death-rate was the highest (35*4) in St-
George's, and the lowest (7"1) in Rusholme. The death-rate for the
whole city was equal to 28'5 per thousand, against 23*5 in the corres-
ponding week last year.
A serious attempt is being made to supply the imperative want of a
large central hospital for the populous districts of South London. It is
proposed to establish a temperance hospital. An influential committee
of the residents is being formed to carry out the undertaking.
James Crombie, pressman, has died at Aberdeen, in fearful agony>
from the effects of having swallowed an ounce of sulphuric acid. The
deceased stated before he died that two men mesmerised him, and com-
pelled him to take the poison?an extraordinary and groundless
hallucination.
The Marquis of Lorne presided lately at the Cannon-street Hotel?
over a combined general meeting of the Federation of Working Men's
Social Clubs and Club and Institute Union, representing over 300 clubs
and about 35,000 working men, to consider the relations between
hospitals and the working classes.

				

